# Stochastic Behavior of Phase Synchronization Index and Cross-Frequency Couplings in Epileptogenic Zones during Interictal Periods Measured with Scalp dEEG

**DOI:** 10.3389/fneur.2013.00057

**Published:** 2013-05-16

**Authors:** Ceon Ramon, Mark D. Holmes

**Affiliations:** ^1^Department of Electrical Engineering, University of WashingtonSeattle, WA, USA; ^2^Department of Bioengineering, Reykjavik UniversityReykjavik, Iceland; ^3^Department of Neurology, University of WashingtonSeattle, WA, USA

**Keywords:** epilepsy localization, dEEG, phase synchronization, stochastic behavior of EEG, cross-frequency couplings

## Abstract

The stochastic behavior of the phase synchronization index (SI) and cross-frequency couplings on different days during a hospital stay of three epileptic patients was studied for non-invasive localization of the epileptogenic areas from high density, 256-channel, scalp EEG (dEEG) recordings. The study was performed with short-duration (0–180 s), seizure-free, epileptiform-free, and spike-free interictal dEEG data on different days of three subjects. The seizure areas were localized with subdural recordings with an 8 × 8 macro-electrode grid array and strip electrodes. The study was performed in theta (3–7 Hz), alpha (7–12 Hz), beta (12–30 Hz), and low gamma (30–50 Hz) bands. A detrended fluctuation analysis was used to find the long range temporal correlations in the SI that reveals the stochastic behavior of the SI in a given time period. The phase synchronization was computed after taking Hilbert transform of the EEG data. Contour plots were constructed with 20 s time-frames using a montage of the layout of 256 electrode positions. It was found that the stochastic behavior of the SI was higher in epileptogenic areas and in nearby areas on different days for each subject. The low gamma band was found to be the best to localize the epileptic sites. Also, a stable higher pattern of SI emerged after 60–120 s in the epileptogenic areas. The cross-frequency couplings of SI in theta–gamma, beta–gamma, and alpha–gamma bands were decreased and spatial patterns were fragmented in epileptogenic areas. Combinations of an increase in the stochastic behavior of the SI and decrease in cross-frequency couplings are potential markers to assist in localizing epileptogenic areas. These findings suggest that it is possible to localize the epileptogenic areas non-invasively from a short-duration (∼180 s), seizure-free and spike-free interictal scalp dEEG recordings.

## Introduction

In previous studies (Ramon and Holmes, 2012, 2013), we have demonstrated that the stochastic behavior of the phase synchronization index (SI) derived from the high density (256-channel) scalp EEG data has a potential to localize the epileptic sites in human subjects. A 256-channel scalp EEG data is often referred as dEEG or hdEEG data. These previous studies were limited either to a single day (Ramon and Holmes, 2012) or for multiple days but in a single EEG band of low gamma frequencies (Ramon and Holmes, 2013). The present work is an extension and a comprehensive summary of the previous work on multiple days in four EEG bands, viz., theta (3–7 Hz), alpha (7–12 Hz), beta (12–30 Hz), and low gamma (30–50 Hz) and it confirms our previous results that the stochastic behavior of the SI in the low gamma band was best suited to localize the epileptic sites derived from a short-duration (∼180 s) interictal scalp dEEG data.

The EEG phase synchronization plays an important role in studying the network connectivity of different regions of the brain under normal and diseased states, including various forms of epileptic seizures (Varela et al., 2001; Baier et al., 2012; Lang et al., 2012; Palmigiano et al., 2012). Local and long rage connectivity can be studied with EEG phase synchronizations (Varela et al., 2001). An increase in the phase synchronization has been observed in epileptogenic zones (Schevon et al., 2007; Warren et al., 2010) and, also, the connectivity patterns are different in epileptogenic zones as compared with other cortical zones not involved in epileptic activities (Varotto et al., 2012). Phase synchronization also influences the genesis of epileptic activity. From invasive recordings it has been observed and suggested that there is an increase in the phase synchronization activity in the epileptogenic regions of the brain and these regions are functionally isolated from the surrounding regions of the brain (Warren et al., 2010). It has also been observed with magnetoencephalogram (MEG) recordings in epileptic patients that there were fluctuations in synchrony between neighboring cortical networks (Dominguez et al., 2005).

The phase synchronization also randomly fluctuates with time. The stochastic behavior of these random fluctuations can be studied with detrended fluctuation analysis (DFA) which is, often, used to study long range temporal correlation (LRTC) in a time series data, such as, EEG data (Peng et al., 1995; Hardstone et al., 2012). It has been shown by Linkenkaer-Hansen et al. (2005) that DFA exposes LRTCs that are characteristic of epileptogenic neocortical networks, the areas where epilepsy begins. We examined the stochastic behavior of the SI on different days derived from a short-duration (∼3 min) interictal dEEG data. Our results show that the stochastic behavior of the SI is higher in the vicinity of the epileptogenic zones and possibly maybe useful to localize the epileptic sites. Here, the stochastic behavior of the SI or LRTC of SI is used in a synonymous fashion.

The brain also exhibits oscillatory activity in various frequency bands. The cross-frequency coupling, where one band modulates the activity of a different band is a very powerful tool to study the oscillatory activity of the brain. It has been observed with subdural recordings that the power of the high gamma (50–100 Hz) band is phase locked to theta oscillations (Canolty et al., 2006). The cross-frequency related phase synchrony among different bands in the frequency range of 3–80 Hz has also been observed in human MEG data (Palva et al., 2005) and also in scalp EEG data (Friese et al., 2012). We also found that during object naming tasks measured with dEEG data there was some coupling in the theta–gamma band (Ramon et al., 2009). This was observed by plotting the difference in phase synchronization between two EEG bands over a time period of 3 s. A similar analysis is used here to examine stochastic behavior of cross-frequency couplings. We found that cross-frequency couplings decreased in theta–gamma, beta–gamma, and in alpha–gamma bands. Also, some complex spatial patterns were observed. These results can also be used as an additional marker to non-invasively localize the seizure onset areas from interictal scalp EEG data.

## Materials and Methods

### EEG data of patients

Our procedures for data collection and analysis have been described previously (Ramon et al., 2008; Ramon and Holmes, 2012, 2013). Only a brief summary is given here. Epileptic seizure areas in patients were localized with intracranial subdural EEG (ECoG) recordings with 8 × 8 contact grid electrodes and also with strip electrodes. The electrodes on the grid had an exposed surface area defined by 2.3 mm diameter and with a center-to-center, inter-electrode separation of 1.0 cm (Johnson et al., 2012). The strips had the same size electrodes with the same inter-electrode separation. Prior to this during pre-surgical evaluations, high density 256-channel scalp EEG data was collected continuously for 7–12 days. The data was collected with an EEG system developed by Electrical Geodesics, Inc. (Eugene, OR, USA). The electrode caps were filled with a conducting gel with an effective diameter of ∼1.0 cm. For an adult head, from the center of one electrode to the other, the inter-electrode separation was ∼2.0 cm or less (Tucker, 1993). The data was collected with a sampling rate of 250 Hz, i.e., the time difference between each sample was 4 ms. We used data of three adult subjects. All were candidates for resection surgery and after surgery they were cured of the epileptic seizures. Subjects were not on any medication during dEEG and ECoG monitoring. All data were collected at the Regional Epilepsy Center, University of Washington under the authorized human subjects protocol.

For each subject, we selected data on three randomly selected days for analysis. For a given day, approximately, 10 min long, seizure-free and spike-free data from each patient during sleep was selected for analysis. The selected data sets were not in close proximity to seizures. Out of this, a continuous 3 min long data was randomly selected and imported into MATLAB for further analysis. The analysis was repeated for other two randomly selected days of the data.

### Computations of phase synchronization index

The raw EEG data was normalized to their common averaged reference and then filtered using a FIR bandpass filter in the appropriate EEG band. Excessively noisy channels were eliminated by replacing them with the averages of their neighbors. For each subject, there was only one noisy channel. The 60 Hz power line artifact was eliminated with a matching pursuit filter (Gratkowski et al., 2006).

The synchronization between a pair of channels was inferred from a statistical tendency to maintain a nearly constant phase difference over a given period of time even though the analytic phase of each channel may change markedly during that time-frame (Freeman and Rogers, 2002). The Hilbert transform was applied on the pairs of EEG traces with a stepping window which is long enough to encompass at least two cycles of the lowest frequency in a given band. For example for the low gamma (30–50 Hz) band, the lowest frequency will be 30 Hz and based on that we selected a window of 80 ms. The analysis was repeated by stepping the window at 8 ms intervals, i.e., two digitized points. Similarly, the size of the stepping window was selected for other EEG bands while the step size of 8 ms was kept the same. The window sizes selected for theta, alpha and beta bands were, 680 ms, 300 ms, and 170 ms, respectively. The SI was computed for each pair of EEG traces.

The phase of the analytic signal has a sawtooth pattern which is unwrapped to produce a cumulative linear phase of the signal. The phase difference between the two channels was computed by subtracting the phase of one channel from the other. This phase difference was then used to determine the SI. The mathematical techniques for computing synchronization indices are given in detail elsewhere (Tass et al., 1998; Freeman and Rogers, 2002). We computed synchronization indices based on Shanon entropy function (Tass et al., 1998). Phase locking, i.e., synchronization between the phases of two signals within a stepping window was given by Shanon entropy function, *e*(*t*), defined as:

(1) $$e\left(t\right)=-\overset{N}{\underset{i=1}{\sum }}p_{i}\ln p_{i}$$

where *p_i_* was the relative frequency of finding the phase difference modulus of 2π in the *i*th bin. The function *e*(*t*) varied between zero and its maximum value of *e*_max_ = ln *N*. We used 100 bins (*N* = 100) for the phase difference in a given stepping window. This phase locking, *q(t)*, was normalized and is represented as:

(2) $$q\left(t\right)=\frac{e_{\max}-e\left(t\right)}{e_{\max}}$$

The *q*(*t*) has a value of zero for uniform distribution of phase differences and a value of one for a spike or delta distribution of phase differences between two signals. This *q(t)* is also called phase syncronization index (SI).

**Figure 1:**
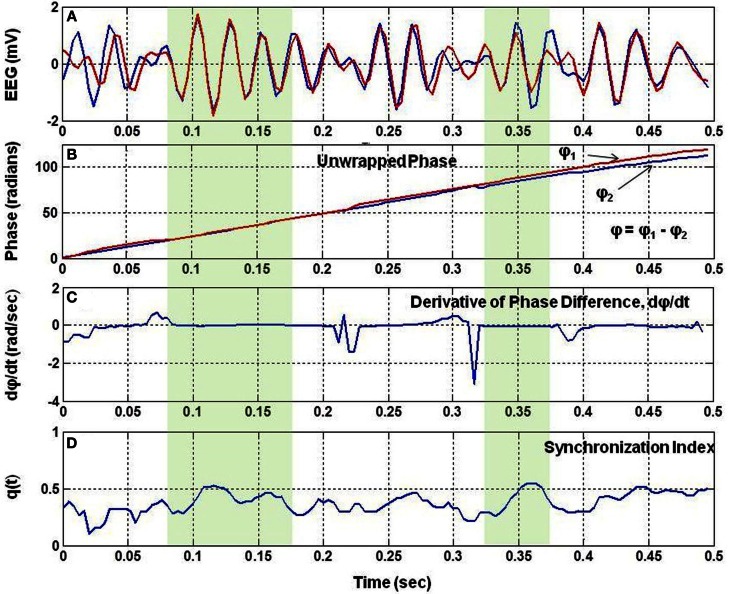
**The analysis of phase synchronization between two EEG signals**. **(A)** A short segment of two EEG traces, **(B)** unwrapped phases, Φ_1_ and Φ_2_ after taking Hilbert transform of EEG signals, **(C)** time derivative, *d*Φ/*dt*, of the phase difference, Φ = Φ_1_−Φ_2_, of two EEG signals, and **(D)** phase synchronization index, *q(t)*, between the two EEG signals. Notice that *q(t)* is high when *d*Φ/*dt* is nearly zero.

The Figure 1 shows a small segment of EEG traces for two nearby channels. It also shows the unwrapped phases, Φ_1_ and Φ_2_, of the two signals, the time derivative of the phase difference, *d*(Φ)/*dt*, and the *q*(*t*). The phase difference is defined as: Φ = Φ_1_−Φ_2_. There are two regions marked with light-green color shade where *d*Φ/*dt* is nearly zero and the *q*(*t*) is high. This shows that in the shaded regions, the two EEG signals are almost in phase synchronization and due to this value of *q*(*t*) is high.

**Figure 2:**
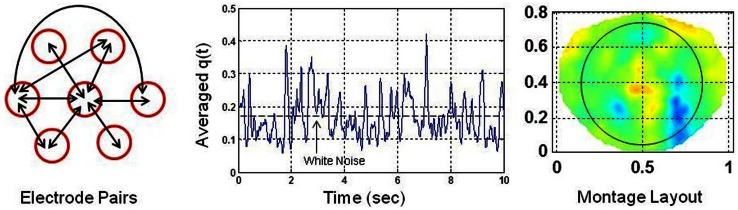
**(Left) each electrode is paired with six nearby electrodes for computation of phase synchronization indices**. There are 21 possible combinations. Few of them are shown by arrows. (Middle) average of phase synchronization indices over 21 combinations of electrode pairs. The phase synchronization index of the white noise is shown with a dashed line. (Right) montage layout of 256 electrodes above the head for plotting purposes. The nose is on the top. The ellipse encloses the area covered by electrodes above the eye level but excludes the electrodes on the forehead.

A global SI was also computed for each electrode by pairing it with the nearby six electrodes. There were 21 combinations of electrode pairs for each given electrode. The *q*(*t*) was averaged over these electrode pairs for each given electrode. The averaged *q*(*t*) represents the local cortical connectivity approximately over a circular area defined by a 4.0 cm diameter which is based on inter-electrode separation of 2.0 cm in the geodesic net used for collecting dEEG data (Tucker, 1993). Figure 2 shows the electrode pairs, the global SI and a montage layout of 256 electrode positions for plotting purposes. The nose is on the top, back of the neck is at the bottom, left of the subject is on the left side of the plot and right side of the subject is on the right side of the plot. The ellipse, roughly covers the electrodes above the eye level including occipital areas, but, excludes the electrodes on the forehead. The horizontal and vertical axes for plots are in normalized length units.

Only few possible electrode pairs, out of possible 21 pairs are shown by the double headed arrows in Figure 2. The averaged *q*(*t*) given in Figure 2 is slightly less than the *q*(*t*) given in Figure 1 for a single electrode pair. In general, there is always some spatial decoherence involved between two electrode pairs (Freeman and Rogers, 2002). Thus, it is anticipated that by averaging over 21 electrode pairs, the averaged SI will be less than the SI of an individual electrode pair. However, it is still above the synchronization level of the white noise (Tass et al., 1998). Following the procedures given in Tass et al. (1998), a Gaussian white noise signal was generated, filtered and the SI was computed. The mean value of the SI of the white noise was found to be 0.18 with 95% confidence interval. The noise level is marked as a dashed line in the middle plot in Figure 2. The averaged *q*(*t*) in Figure 2 shows sustained levels of phase synchrony which is interrupted by dips below the noise level. Hereafter, the averaged *q*(*t*), for simplicity, is called the phase syncronization index (SI).

### Computations of stochastic behavior

We used DFA to compute the stochastic behavior of the SI. The cumulative sum of each channel was calculated. This sum was divided into windows of 10 s length, i.e., windows were of 10, 20, 30, …, 170 and 180 s length. Within each window, a linear fit was found and the cumulative sum was detrended. Next, the root-mean-squared (RMS) fluctuation of this detrended sum was calculated. The median fluctuation at each window size was taken. The log of this median fluctuation was plotted against the log of the window size, and a linear fit was found. The slope of this linear fit, denoted as, α, is the result of the DFA which can be expressed as (Hardstone et al., 2012):

(3) $$F\left(L\right)\propto L^{\alpha }$$

where *F*(*L*) is a fluctuation function and *L* is the window length. This is also called LRTC (Peng et al., 1995) of a given time series data, such as, EEG or SI which is derived from EEG. For simplicity, we will refer LRTC of SI as the stochastic behavior of the SI. The stochastic behavior of the SI, i.e., α, was computed for each channel as explained above. Color intensity plots of α were constructed using a montage layout of 256 electrode positions given in Figure 2.

The exponent, α, in Eq. 3 is also related to the power law representation of random walk processes after a walk of length *L* (Hardstone et al., 2012). This exponent, α, represents many properties of a time series data. If 0 < α < 0.5, the slope is negative and the time series sequence is anticorrelated or negatively correlated. If α ≃ 0.5, the time series sequence is an uncorrelated white noise. If 0.5 < α < 1, the time series sequence has LRTCs. If α ≃ 1.0, the time series sequence is a 1/*f* pink noise, where *f* is the frequency of the signal. If α > 1.0, the time series sequence is non-stationary, random walk like and has strong correlations which are not of a power law form. A special case is for α ≃ 3/2 which represents that the time series sequence is a Brownian noise. In general, the EEG signal is non-stationary while the white noise is stationary.

## Results

### Spatiotemporal plot

**Figure 3:**
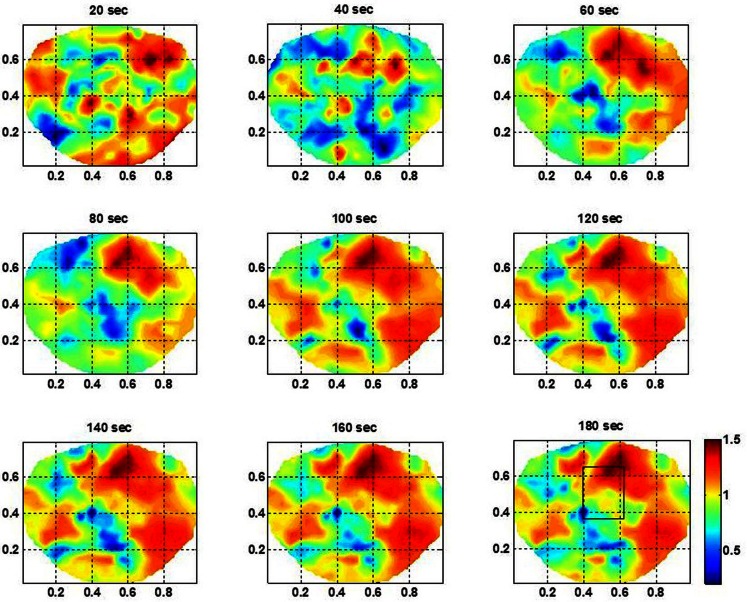
**The spatiotemporal plots of the stochastic behavior of the phase synchronization index (SI) for the subject #1 in the low gamma band**. These plots, above the head are with 20 s intervals over a period of 3 min. The seizure area is marked with a rectangle in the plot at 180 s. Notice that the stochastic activity is higher in the seizure area and becomes stable and noticeable after the 60 s time-frame.

For the subject #1, the spatiotemporal plots of the stochastic behavior of the SI are given in Figure 3. A rectangle marks the location of the seizure area as mapped with invasive subdural grid and strip electrode recordings. The subdural grid was near to the midline covering the right frontal, parietal and temporal areas of the brain. Two strip electrodes were covering the medial frontal and medial parietal areas of the brain. This subject had seizure in central parietal area and frontoparietal midline areas. The scalp dEEG electrodes also showed seizure activities in the same area. These seizures were more toward the right side of the brain from the midline. In Figure 3, the rectangle is shown in the 180th second of the plot. A stable pattern of stochastic behavior begins to emerge from 60 s onward. Similar plots were also constructed for other two subjects which are not shown here. The color bar gives the LRTC values, i.e., α values. Within the seizure area, the values of α are in the range of 1–1.5 indicating a strong LRTC which are not of a power law form.

### Subject #1

**Figure 4:**
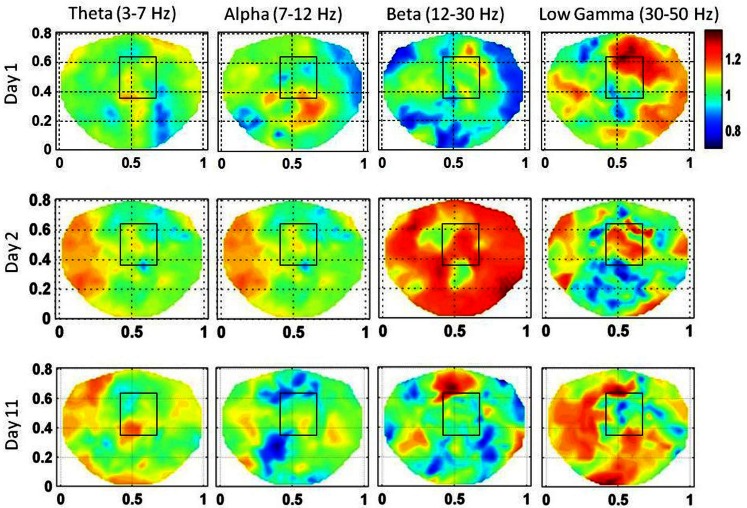
**The stochastic behavior of the phase synchronization index (SI) for the subject #1 in all EEG bands for three different days**. Columns are the EEG bands and rows are the days. Notice that for the low gamma band, stochastic activity is higher in and near to the seizure area on all 3 days while for the beta band, it is higher on the second and the eleventh day. This behavior is not strong for theta and alpha bands.

**Figure 5:**
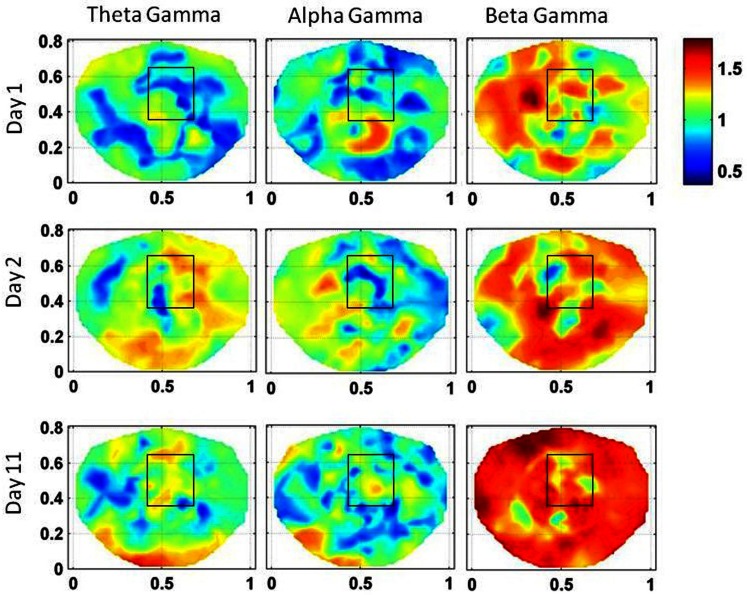
**Stochastic behavior of coss-frequency couplings for the subject #1 on three different days**. In the seizure area, it seems to be fragmented and with spatial dipolar and quadrupolar patterns. These patterns are more pronounced in the low gamma band.

The stochastic behavior of the SI and cross-frequency couplings for the subject #1 are given in Figure 4 and Figure 5, respectively. For this subject, as shown in Figure 3, 60–80 s duration of dEEG data was sufficient to localize the epileptic site. The analysis was done for the dEEG data collected on the first, second, and the eleventh day of the hospital stay. The seizure area is marked with a rectangle that was determined with invasive subdural grid and strip electrode recordings. The stochastic behavior of the SI in the seizure area is higher as compared with nearby surrounding areas on all 3 days in the low gamma band. Refer to Figure 4. It is strongest on the first day and fragmented on other days, but, still with hot spots within and near to the seizure area. In the beta band, on the first day, the stochastic activity is very low in the seizure area. On the second day, it is wide spread, including in the seizure area. On the eleventh day, there is some higher activity at the upper edge of the seizure area. The theta and alpha bands do not show any hot spots in the seizure area.

The stochastic behavior of the cross-frequency couplings is given in Figure 5. In general, it is fragmented and less as compared with surrounding areas in all the bands and on all of 3 days, i.e., first, second, and the eleventh day. It is more noticeable in the beta–gamma coupling. The α values for theta–gamma and alpha–gamma couplings are in the range of 0.5–1.2. In comparison, the α values for beta–gamma couplings are higher and are in the range of 1.2–1.6. In Figure 4, there are other hot spots outside the seizure in all of these plots which could be related to the spread of the seizure activity in a larger area or due to the some other processes in the brain, such as, spontaneous brain activity. This makes it difficult to localize the seizure area from the stochastic behavior of the SI alone in different EEG bands. However, combining with cross-frequency couplings, it becomes feasible to localize the seizure areas.

### Subject #2

**Figure 6:**
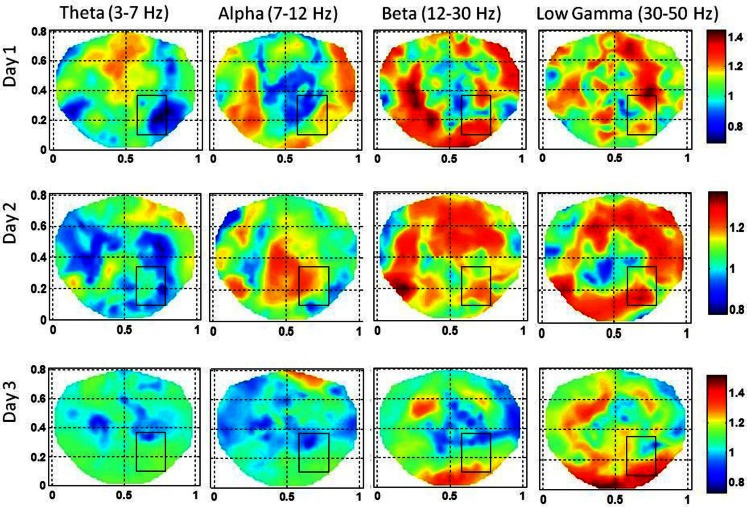
**The stochastic behavior of the phase synchronization index (SI) for the subject #2 in all EEG bands for three different days**. Notice that for the low gamma band, stochastic activity is higher in and near to the seizure area on all 3 days while for the beta band, it is higher on the second and the third day. This behavior is not strong for theta and alpha bands.

This subject had a complex seizure pattern spreading from right mid temporal area to the right occipital area. The spread of the seizure activity was seen in the grid and strip electrode recordings. The stochastic behavior of the SI is given in Figure 6 for the first, second, and the third day of the hospital stay. For this subject, 100 s long EEG data was needed to find a stable pattern in the stochastic activity of the SI. The gamma band exhibits hot spots in the seizure are and also in the vicinity of seizure area on all 3 days. Also, there are hot spots in the beta band on the first 2 days in the seizure area. On the third day, the hot spots are toward the lower edge of the rectangle, very close to the occipital area. As compared with low gamma and beta bands, the activity in alpha and theta bands is less intense. For the first 2 days, the alpha band exhibits some low intensity activity in the seizure area and on the third day it is missing. The theta band does not show any activity in the seizure area.

**Figure 7:**
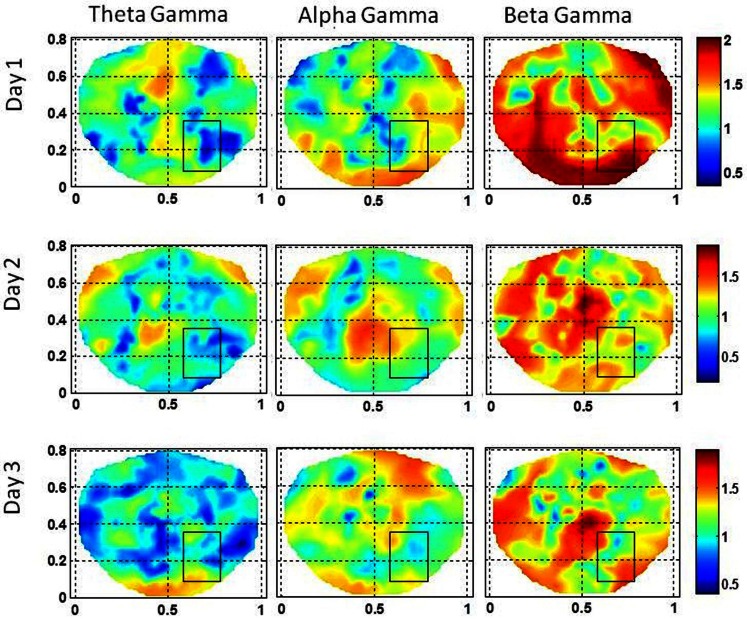
**Stochastic behavior of coss-frequency couplings for the subject #2 on three different days**. In the seizure are, it seems to be fragmented with complex spatial patterns for beta–gamma couplings. These cross-frequency couplings in theta–gamma and in alpha–gamma bands are very low.

The stochastic behavior of cross-frequency couplings is given in Figure 7 for the subject #2. For the beta–gamma coupling within the seizure area, the activity is fragmented and exhibits a dipolar and quadrupolar patterns on all 3 days. There is also significant activity outside the seizure area on the first day which could be due to the spreading of the seizure activity and/or due to spontaneous brain activity. On the first day, there is some low level stochastic activity in the alpha–gamma coupling which is reduced significantly on the following 2 days. The theta–gamma coupling is absent in the seizure area on all 3 days. Within the seizure area the values of α are in the range of 1.0–2.0 for alpha–gamma and beta–gamma couplings. In contrast for theta–gamma couplings, the values of α are in the range of 0.5–1.0. These plots show that the stochastic behavior of the cross-frequency coupling in the beta–gamma band is highest as compared with theta–gamma and alpha–gamma couplings.

### Subject #3

**Figure 8:**
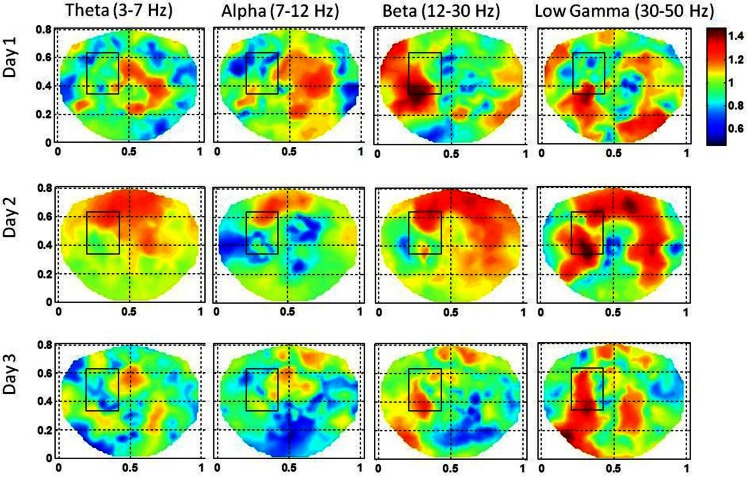
**The stochastic behavior of the phase synchronization index (SI) for the subject #3 in all EEG bands for three different days**. Notice that for the low gamma band, stochastic activity is higher in and near to the seizure area on all 3 days while for the beta band, it is higher on the first and the second day. This behavior is not strong for the alpha band. However, the theta band does show some hot spots on the second day in the seizure area.

This subject had seizures in the left front temporal area with slow bilateral spread which were observed in subdural grid electrodes, strip electrodes and also in scalp dEEG recordings. The subdural grid was placed on left frontal and temporal areas and electrode strips were placed on left medial temporal, lateral frontal and occipital areas. The stochastic behavior of the SI is given in Figure 8 for the first, second, and the third day of the hospital stay. For this subject, 100–120 s long EEG data was needed to find a stable pattern in the stochastic activity of the SI. The gamma band exhibits hot spots in the seizure area on all 3 days. On the first day, the hot spot is observed only at the lower edge of the seizure area rectangle. The hot spots in the vicinity of right occipital area are probably due to the spread of the seizure activity. These seizures related hot spots are much stronger on the second day and also there is widespread activity in the frontal and right temporal areas which was also observed in strip electrodes. The low gamma band activity is very strong in the seizure area marked by the rectangle and also outside, below the rectangle. The beta band exhibits hot spots in the seizure area on the first 2 days while on the third day it is very subdued. The alpha band activity within the seizure area is absent on the first and the third day while some low level activity is present on the second day. Patterns similar to the alpha band activity are also present in the theta band activity. No theta band activity in the seizure area on the first and the third day. There is a widespread activity in the frontal area, including the seizure area on the second day in beta and low gamma bands. This could be related to bilateral spread as observed in invasive grid and strip electrode recordings.

**Figure 9:**
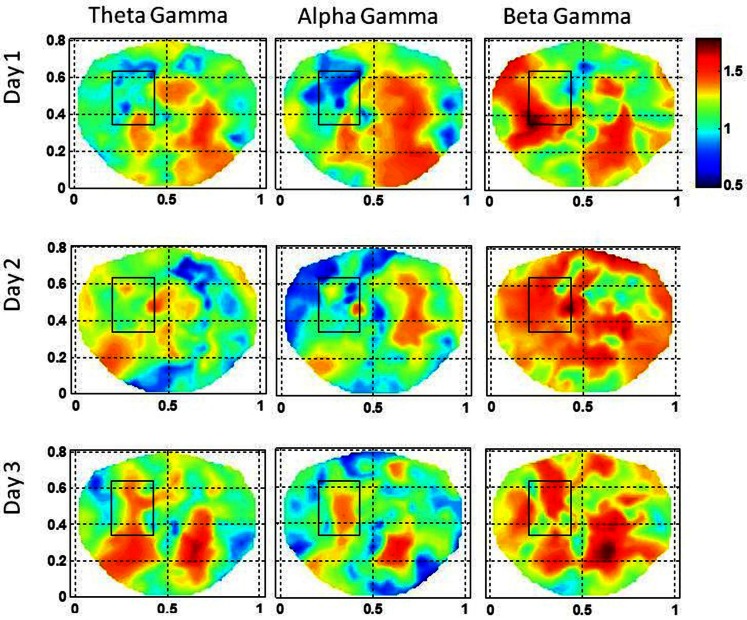
**Stochastic behavior of coss-frequency couplings for the subject #3 on three different days**. In the seizure are, it seems to be fragmented with complex spatial patterns for the beta–gamma coupling. These cross-frequency couplings are very low in theta–gamma and in alpha–gamma bands.

The stochastic behavior of cross-frequency couplings for the subject #3 is given in Figure 9. The stochastic behavior of the beta–gamma coupling within the seizure area is fragmented and exhibits complex spatial patterns on the first and the third day. On the second day, the stochastic behavior of the beta–gamma coupling is spread in a large area on the left side including the seizure area. The stochastic behavior of the alpha–gamma coupling is absent in the seizure area on the first and the second day, but some activity in visible on the third day. The stochastic behavior of the theta–gamma coupling is absent in the seizure area on the first day, slightly visible on the second day and becomes stronger on the third day.

## Discussions

For the subject #1, the stochastic behavior of the SI in the low gamma band (Figures 3 and 4) seems to show some higher patterns of activity in and near to the seizure area on all 3 days. In addition, the stochastic behavior of the beta–gamma coupling is fragmented in the seizure area. Also, behaviors of other cross-frequency couplings are also weaker in the seizure area with respect to the nearby areas. For this subject a stable pattern of stochastic activity (Figure 3) begins to appear from 60 s onward and thus, 80 s long interictal dEEG data was enough to localize the epileptic site.

For the subject #2, both the beta and the low gamma bands had the higher stochastic activity of the SI in and near to the seizure area and the stochastic behavior of the coss-frequency couplings was fragmented and relatively decreased. For this subject, 100 s long interictal dEEG data was needed to find stable patterns of stochastic activity in the seizure area.

For the subject #3 also, both the beta and the low gamma bands had the higher stochastic activity of the SI in and near to the seizure area and the stochastic behavior of the coss-frequency couplings was fragmented and relatively decreased. For this subject, 100–120 s long interictal dEEG data was needed to find stable patterns of stochastic activity in the seizure area. An interesting feature to note is that for subjects #2 and #3 on the same days when the beta band activity is higher, in comparison, the low gamma band activity is lower.

From these results and from our previous work (Ramon and Holmes, 2012, 2013) one can conclude that in the seizure areas and also in the vicinity of seizure areas, stochastic behavior of the SI in beta and low gamma bands are higher and also the stochastic behavior of cross-frequency couplings has decreased and spatial patterns are fragmented. Our results also suggest that the seizure related stochastic activity is present on a continuous basis in the interictal scalp EEG data which, possibly, could be useful for non-invasive localization of the epileptic sites. These are our preliminary results and show a promise that these have a potential to localize epileptic sites from a short-duration (1–3 min), seizure-free, high density (256-channel) scalp EEG data. Further studies with more subjects are needed to substantiate these findings.

The SI is a time series sequence and in the seizure area, the values of α are in the range of 0.8–2.0 for beta and low gamma bands. Thus, in these two bands, SI exhibits a LRTC or strong correlations and also exhibit some properties of Brownian noise within the seizure area. In contrast, theta and alpha bands have lower values of α in the range of 0.4–1.2. This would suggest that the stochastic behavior of the SI in theta and alpha bands is an uncorrelated white noise in some parts of seizure area and in other parts, it has LRTC or strong correlations. Based on this, one can further infer that beta and low gamma bands in combination with cross-frequency couplings will be a better choice for localization of the epileptic sites.

The SI for a given electrode is averaged with the SI of nearby six electrodes. This averaged SI represents the local interconnected activity of cortical neurons. The stochastic behavior of the SI showed in Figures 3–9 is often spread in large areas including the seizure areas. It could possibly represent the local and global connectivity of cortical neurons in the seizure areas as well as normal parts of the brain in the vicinity of the seizure area. Epileptic and interconnected normal cortical neurons, both play an important role in the genesis and spread of the seizure activity. It is also possible that the stochastic behavior of the SI in interictal periods is spread in an area larger than the seizure area. These concepts need to be further examined with seizure modeling and patient studies.

An increase in fluctuations in the order parameter related to brain synchronization has also been observed and can be used as precursors for identification of epileptic seizures (Velazquez et al., 2011). The order/disorder parameter can also be used as a measure of EEG phase transition states of the brain. In view of these previous studies, our results based on the stochastic fluctuations in the phase synchronization indices are plausible.

## Conflict of Interest Statement

The authors declare that the research was conducted in the absence of any commercial or financial relationships that could be construed as a potential conflict of interest.
